# Link between structural risk factors for adverse impacts of COVID-19 and food insecurity in developed and developing countries

**DOI:** 10.1007/s10668-022-02749-x

**Published:** 2022-11-09

**Authors:** Luisa Marti, Rosa Puertas

**Affiliations:** Group of International Economics and Development, Universitat Politècnica de València, Camino de Vera S/N, 46022 Valencia, Spain

**Keywords:** Food insecurity, COVID-19, Contingency tables, TOPSIS

## Abstract

COVID-19 has had serious consequences for world food security; lockdowns and social distancing have led to changes in global food value chains, primarily affecting the poorest of the planet. The aim of this research is to analyse the relationship between food insecurity and the structural risk factors for adverse impacts of COVID-19. To that end, 12 contingency tables are constructed to identify the association between the pillars of the food insecurity index and the INFORM COVID-19 Risk Index. We use the Gamma coefficient as a measure of association. In addition, this paper proposes a synthetic index produced by applying the TOPSIS method, using the pillars of the two aforementioned indices (criteria) to establish a ranking of 112 countries (alternatives) ordered from highest to lowest risk faced in the key year of the pandemic, 2020. The results show that the two problems are connected, indicating to international organizations that countries with worse food insecurity will suffer more serious consequences from extreme situations such as the one experienced during the pandemic. The ranking established directs international organizations' attention to countries such as Haiti, Zambia and Burundi, highlighting their greater need for an injection of financial aid than other emerging economies. Conversely, Switzerland is the country with the lowest combined risk.

## Introduction

In times of economic prosperity, little attention is given to the vulnerability of food systems. However, in times of political or socio-economic turmoil, national efforts to strengthen such systems determine the impact on overall levels of food security (EIU, 2020). COVID-19 has had major implications for global nutrition security, as lockdowns and social distancing requirements have disrupted global food value chains, primarily affecting the poorest of the planet (Laborde et al., 2020; Morton, 2020; Pereira & Oliveira, 2020; Swinnen & McDermott, 2020). Major intergovernmental groups warn that if immediate measures are not taken, we may witness a global food emergency (European Commission, 2020).

According to the World Food Summit (1996), food security is defined as the state in which people have, always, physical, social and economic access to sufficient nutritious food to meet their dietary needs for an active and healthy life. Due to its importance, it is one of the key aims of the Sustainable Development Goals (SDGs), specifically SDG 2 (zero hunger), which addresses environmental sustainability and food security to ensure better nutrition and healthy lives (Campi et al., 2021). Developing countries have to deal with hunger caused in part by poor governance, conflict and climate change. In this context, Otekunrin et al. (2020) assess Africa's readiness to reach the zero hunger goal by 2030 in the wake of the COVID-19 pandemic; their results indicate that the high prevalence of undernourishment, stunting and child wasting present significant challenges, hampering the achievement of the zero hunger target. In this research, the Global Food Security Index (GFSI) produced by the Economist Intelligence Unit is used to measure the drivers of food security in 112 countries with widely differing economic profiles (EIU, 2020). This index includes the pillars of food affordability, availability, quality and safety, along with natural resources and resilience.

Another problem currently afflicting countries relates to the consequences of COVID-19, with the most economically vulnerable countries lacking sufficient resources to tackle them (Poljansek et al., 2020). Also relevant are vulnerability issues relating to the proportion of the population at increased risk of severe COVID-19 disease, inequality, economic dependence, uprooted people, gender-based violence, health conditions, food security, the capacity of health systems specific to COVID-19, governance and access to health care. In response to this situation, an index has been developed that offers an international comparison; namely, the INFORM COVID-19 Risk Index (ICRI), published in 2020 by the European Commission (Joint Research Centre). The ICRI is composed of 3 pillars (Hazard and exposure, Vulnerability and Lack of coping capacity. It primarily identifies structural risk factors, that is, factors that were present before the outbreak of the pandemic. The resulting ranking of countries provides information about their specific needs, facilitating the decision-making of humanitarian organizations.

Based on an analysis of the ICRI, Moldes-Anaya et al. (2021) reveal regional variation in terms of COVID-19 risk, underscoring the importance of public policy strategies to address the impacts of coronavirus. Qazi et al. (2021) have used the index to show that the risk ratings associated with pre-COVID-19 disasters risk and COVID-19 risk are statistically strongly correlated.

The aim of this research is to analyse the relationship between food insecurity and the risk of not having the capacity to deal with all the health and humanitarian consequences of COVID-19, thus requiring international assistance. To compute the food insecurity index (FII), we take the inverse of the GFSI. We then develop a synthetic index (SI) covering both aspects, to get a global overview of the problem. The study is carried out using a sample of 112 countries for 2020. The results provide answers to the following research questions:

Q1. Is there any association between the pillars of the ICRI and those of the FII?

Q2. Does the ranking of the countries based on the new SI differ from the individual ICRI and FII rankings?

In the literature, there are studies focused solely on food insecurity (Allee et al., 2021; Alnafissa et al., 2021; Caccavale & Giuffrida, 2020; Odhiambo et al., 2021; Zidouemba et al., 2020); on structural risk factors for COVID-19 impacts (Arsalan et al., 2020; Huang et al., 2020; Moldes-Anaya et al., 2021); as well as studies focusing on both aspects jointly but for a single country such as Nigeria (Amare et al., 2021), Bangladesh (Ahmed et al., 2021), Mali (Adjognon et al., 2021), the United Kingdom (Ranta & Mulrooney, 2021), Belgium (Vandevijvere et al., 2021), USA (Nagata et al., 2021), India (Mishra & Rampal, 2020), Jordan (Elsahoryi et al., 2020) and Burkina Faso (Zidouemba et al., 2020). In this research, we perform a joint analysis of the ICRI and the FII for a sample of countries with widely differing socio-economic characteristics. The results will be useful to international organizations, allowing them to detect possible shortcomings and patterns of behaviour that can serve as a model for subsequent implementation. The recency of the data and the number of countries analysed mean we can report conclusive results on the factors that drive food security and structural risk factors of the pandemic in these countries.

## Variables and sample

The empirical analysis carried out here focuses on food insecurity and structural risk factors for COVID-19 impacts, using information from components of the FII and the ICRI, both published in 2020. The GFSI includes the pillars of food affordability, availability, quality and safety, along with natural resources and resilience. The objective of this index is to assess which of the 113 nations are most vulnerable to food insecurity, identifying aspects that need special attention. The index is a dynamic quantitative and qualitative benchmarking model constructed from 58 unique indicators that measure the drivers of food security across both developing and developed countries (EIU, 2020). The content of the pillars is detailed below (EIU, 2020):

- Affordability (weight: 32.4%). Measures consumers' ability to buy food, their vulnerability to price shocks and the presence of programmes and policies to support them when shocks occur.
- Availability (weight: 32.4%). Measures the sufficiency of the national food supply, the risk of supply disruption, national capacity to distribute food, and research efforts to boost agricultural output.
- Quality and safety (weight: 17.6%). Accounts for the variety and nutritional quality of average diets, as well as the safety of food.
- Natural resources and resilience (weight: 17.6%). Assesses a country's exposure to the impacts of climate change, its susceptibility to natural resource risks, and how the country is adapting to these risks.

In order to use the pillars in the contingency tables, a categorization must be established by dividing the scores achieved, which range between 0 and 100 in all cases, into levels. Following the criteria set out in the GFSI (EIU, 2020) the categories are: *very good* (+ 80), *good* (60–79.9), *moderate* (40–59.9), *weak* (20–39.9), *very weak* (− 19.9). In this study, the FII of each country “i” has been calculated by transforming both its overall score and the score for each of its pillars as follows:

1 $$ {\text{FII}}_{i} = 100 - {\text{GFSI}}_{i} $$

Thus, countries that are categorized as *very good* with the GFSI index are categorized as *very weak* when constructing the contingency tables, with the same relationship applied to the rest of the levels. This transformation means that countries with more food insecurity register higher scores, which is necessary in order ensure homogeneity with the ICRI and subsequently be able to produce the SI.

**Fig. 1:**
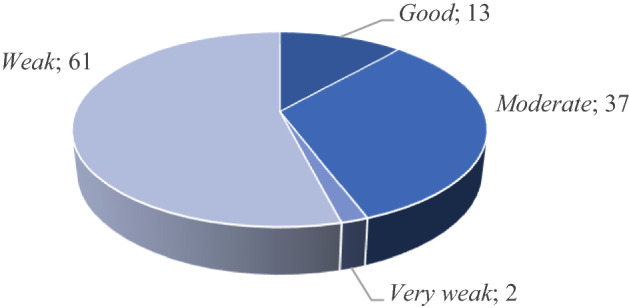
Distribution of economies by level according to the overall FII score

Figure 1 shows an uneven distribution of countries among the levels considered in the FII; the largest group is comprised of countries categorized as *weak* (54% of the total), followed by *moderate* (32.7%).

The ICRI identifies the structural risk factors for COVID-19 impacts for 191 developed and developing economies. The index can help to prioritize preparedness and early response actions to address the primary impacts of the pandemic and identifies countries where secondary effects may have the most critical humanitarian consequences. Its primary area of application is the allocation of resources at the global and regional level accounting for information on risk, that is, where a better understanding of countries' differing situations is important (Poljansek et al., 2020). The ICRI is composed of the following pillars:

- Hazard and exposure: including population density, urban population growth, population living in urban areas, population living in slums, household size, sanitation, drinking water, and hygiene. The hazard and exposure dimension reflects the probability of physical exposure associated with specific hazards. There is no risk if there is no physical exposure, no matter how severe the hazard event is. Therefore, the hazard and exposure dimensions are merged into hazard and exposure dimension. As such it represents the load that the community has to deal with when exposed to a hazard event. The dimension comprises two categories: natural hazards and human-induced hazards, aggregated with the geometric mean, where both indexes carry equal weight within the dimension.
- Vulnerability: including international and internal movement, awareness, trust, proportion of the population at increased risk of severe COVID-19 disease, development and deprivation, inequality, economic dependence index, uprooted people, gender-based violence, health conditions, food security. There are two categories aggregated through the geometric average, socio-economic vulnerability and vulnerable groups. The indicators used in each category are different in time variability and the social groups considered in each category are the target of different humanitarian organizations. If the first category refers more to the demography of a country in general, the vulnerable group category captures social groups with limited access to social and health care systems.
- Lack of coping capacity: including health systems’ capacity specific to COVID-19, governance, access to health care. It is aggregated by a geometric mean of two categories: institutional and infrastructure. The difference between the categories is in the stages of the disaster management cycle that they are focusing on. If the institutional category covers the existence of Disaster Risk Reduction programmes which address mostly mitigation and preparedness/early warning phase, then the infrastructure category measures the capacity for emergency response and recovery.

The data used in ICRI comes from international organisations and academic institutes and is considered to be the most reliable available. INFORM works directly with source organisations to ensure quality and appropriate use of the source data (Poljansek et al., 2020).The overall value of the index and its pillars lie between 0 and 10, with the maximum score of 10 indicating the greatest risk.

**Fig. 2:**
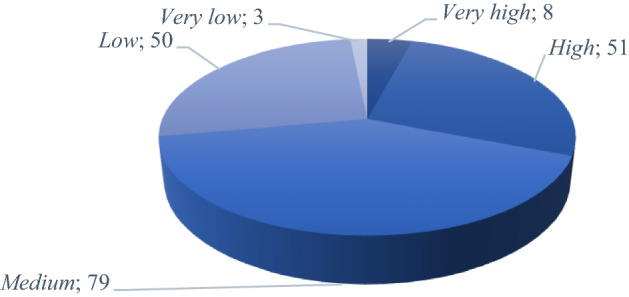
Distribution of economies by level according to the overall ICRI score

ICRI is categorized according to Poljansek et al. (2020) into five levels: *very high* (+ 6.5), *high* (6.4–5), *medium* (4.9–3.5), *low* (3.4–2) and *very low* (− 1.9). Figure 2 depicts the number of countries in each level.

In order to use both indices, it was first necessary to ensure the homogeneity of the sample. As a result, South Korea was removed from the FII as there is no ICRI data for that country. Likewise, 79 nations included in the ICRI were removed as they were not in the FII. Thus, the SI was generated with a total of 112 countries, all characterized by the same pillars; the main statistics of these pillars are presented in Table 1.

**Table 1:** Main statistics

	Mean	Max	Min	SD
*FII*				
Affordability	34.2	81.7	7.8	20.9
Availability	42.8	72.5	18.0	11.6
Quality and safety	32.5	66.9	5.5	18.3
*ICRI*				
Natural resource and resilience	51.0	67.8	26.5	9.0
COVID-19 hazard and exposure	4.3	7.9	2.0	1.7
COVID-19 vulnerability	4.6	8.1	2.2	1.7
COVID-19 lack of coping capacity	3.8	7.6	0.0	2.2

Regarding the mean values for the FII pillars, *Natural resource and resilience* stands out as having the highest value, indicating that exposure to climate change is the greatest risk indicator. As for the ICRI pillars, countries' vulnerability shows a higher mean value than the other two pillars. Except for Portugal, almost all the maximum values correspond to developing countries (Affordability, Malawi; Availability, Yemen; Quality and Safety, Sierra Leone; Natural resource and resilience, Benin; Hazard and Exposure, Congo; Vulnerability, Portugal; Lack of coping capacity, Burundi). Portugal is a developed country where the ageing of the population and other related factors are behind the greater degree of vulnerability and risk (Poljansek et al., 2020).

## Methodology

The degree of association between the pillars of the FII and the ICRI is analysed by constructing contingency tables, a method that dates back to the work by Gale and Shapley (1962) and Becker (1973). In the field of food security or food poverty, several studies have applied the Chi-square test to identify the association between variables in a contingency table (Al-Shabib et al., 2017; Bui & Hoang, 2021; D'Amico et al., 2018; Djekic et al., 2017; Marti et al., 2021; Walaszczyk & Galinska, 2020).

The main objective of this method is to analyse the degree of association of a set of elements with different characteristics, which are represented by categories of the descriptive variables under study. Both the observed and expected frequencies are necessary to perform the $\chi^{2}$ test showing whether the variables considered in the study are independent or not. In this paper, we use the Gamma coefficient as a measure of association. Also known as Goodman and Kruskal's gamma, it is a nonparametric measure of the strength and direction of association between two variables measured on an ordinal scale (Barbiero & Hitaj, 2020). Its value ranges between -1 and 1, with the sign indicating either a direct or inverse relationship between the pillars studied.

The SI has been constructed using Technique for Order of Preference by Similarity to Ideal Solution (TOPSIS) proposed by Hwang and Yoon (1981). In the same context, Ahmadi Dehrashid et al. (2021) used the Fuzzy TOPSIS technique to evaluate the status of food security in rural areas of Iran. In addition, Singh et al. (2021) propose a Fuzzy TOPSIS approach to exploring and ascertaining the socio-economic indicators that the construction of rural roads has impacted. TOPSIS was originally proposed by Hwang and Yoon (1981) as a solution to the problem of how to rank alternatives, based on the concept of the distance of the alternative to the positive ideal solution and the negative ideal solution.

TOPSIS has two main advantages: its mathematical simplicity and considerable flexibility in the definition of the choice set. It consists of six consecutive stages (Karabiyik & Kutlu, 2018):

- The decision matrix must be created (*X*_*ij*_)_*m* × *n*_, with *m* alternatives and *n* criteria. In this study, the criteria are each of the pillars that make up the FII and the ICRI, while the alternatives are the countries under analysis.
- The normalized decision matrix (*r*_*ij*_)_*m* × *n*_ is generated, which represents the relative performance of the alternatives.
- The weighted normalized decision matrix is obtained (*V*_*ij*_ = *w*_*j*_ ‧ *r*_*ij*_)_*m* × *n*_. The weights (*w*_*j*_), which indicate the importance of the criteria, are specified by the decision-maker ($\mathop \sum \nolimits_{j}^{n} w_{j} = 1$). In this study, the same weights are used for each criterion, so as not to introduce any subjectivity into the analysis.
- Positive and negative ideal solutions are detected. In this paper, the ideal solution is identified by maximizing each criterion since we want the most at-risk countries to occupy the top positions in the ranking, while the negative ideal solution is calculated by minimizing the criteria.
- The distance to the positive ideal solution (*A*^+^) and to the negative ideal solution (*A*^−^) is evaluated. The Euclidean distance separating each competing alternative from the positive ideal solution (*S*^+^_*i*_) and negative ideal solution (*S*^−^_*i*_) is measured.
- The relative closeness to the ideal solution for each competing alternative is computed.
  $$ CC_{i} = \frac{{S_{i}^{ - } }}{{S_{i}^{*} + S_{i}^{ - } }},\quad \left( {0 < CC_{i} < 1, \, i = 1,2, \ldots ,m} \right) $$

The preference order of the alternatives is established, according to their relative closeness to the ideal solution. Higher values of relative closeness indicate a higher preference order among alternatives (Lin et al., 2008; Lourenzutti & Krohling, 2016).

## Results and discussion

**Fig. 3:**
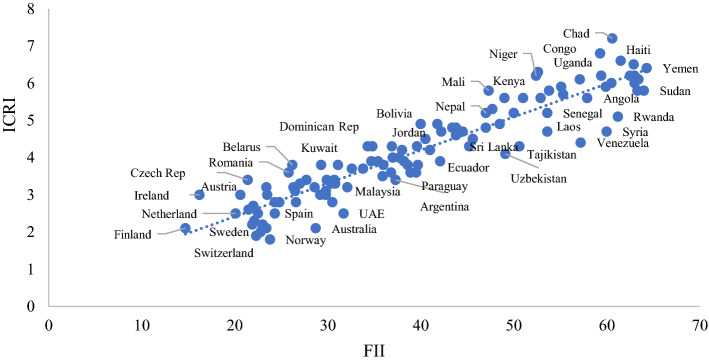
Relationship between FII and ICRI for developed and developing countries

There is a very close relationship between the ICRI and FII overall scores, as shown by the clustering around the trend line in Fig. 3.

The highest scoring nations are characterized as *low income* by the World Bank, while the others all belong to the *high income* category. Thus, the link between the indices could reveals the need to boost aid to the poorest in order to combat the consequences of possible pandemics and achieve a better food security. The pillars that make up the ICRI and the FII more specifically capture the different causes of the structural risk factors for COVID-19 impacts and food insecurity. In this context, the following research questions arise:

Q1. Is there any association between the pillars of the ICRI and those of the FII?

In order to answer this question, 12 contingency tables have been constructed to determine the degree of association between the ICRI and FII pillars.

In this study, a rectangular matrix is constructed, where the rows and columns show the number of countries that register the same level in the two analysed characteristics, constituting the observed frequency. The scores for the pillars of the FII and the ICRI have been transformed into categorical variables with four levels, following the methodology established for each index. Table 2 shows the values of *χ*^2^ and the Gamma coefficient, which indicate the strength and the direction of the connection between the pillars.^1^

**Table 2:** Statistical relationship between FII and ICRI indicators

Indicators with a negative association	*χ*^2^ Test	Gamma
Affordability—Covid-19 hazard and exposure	110.579	− 0.189
	(0.000)	(0.123)
Availability—Covid-19 hazard and exposure	49.056	− 0.353
	(0.000)	(0.007)
Quality and safety—Covid-19 hazard and exposure	88.274	− 0.235
	(0.000)	(0.061)
Natural resources and resilience—Covid-19 hazard and exposure	16.059	− 0.261
	(0.013)	(0.090)

Indicators with a positive association	*χ*^2^ Test	Gamma
Quality and safety—covid-19 vulnerability	67.386	0.214
	(0.000)	(0.012)
Quality and safety—Covid-19 lack of coping capacity	77.492	0.159
	(0.000)	(0.081)
Affordability—Covid-19 vulnerability	85.397	0.151
	(0.013)	(0.075)
Availability—Covid-19 vulnerability	44.354	0.306
	(0.000)	(0.013)
Availability—Covid-19 lack of coping capacity	60.305	0.345
	(0.000)	(0.001)
Affordability—Covid-19 lack of coping capacity	87.423	0.100
	(0.000)	(0.238)
Natural resources and resilience—Covid-19 vulnerability	16.556	0.115
	(0.011)	(0.488)
Natural resources and resilience—Covid-19 lack of coping capacity	22.259	0.164
	(0.004)	(0.216)

*p*-values are shown in parentheses

The results show a significant association between all the pillars of the FII and the ICRI (*p*-value *χ*^2^ test < 0.005**)**. These results suggest that insufficient food and a lack of resources are factors that exacerbate the negative consequences of the pandemic (*Q1*). In addition, according to the gamma coefficient, a symmetrical measure of association, all the FII pillars are positively related to vulnerability and lack of capacity. This indicates that countries facing more difficulties concerning the availability and quality of food, such as Benin, Burundi or Zambia, are more likely to have greater structural risk factors for COVID-19 impacts due to their major economic and healthcare weaknesses. The COVID-19 Hazard and exposure pillar is negatively related to availability, food quality and natural resources (the association is not significant in the case of affordability). Thus, the Netherlands and Switzerland, which have a high level of food security, are exposed to a medium level of risk regarding lack of capacity to cope with COVID-19, with an inverse relationship found between the two indicators. These results are in line with those reported by Mahbub and Rahman (2021), who find that the pandemic has spread more rapidly in economically prosperous countries, although it is deadlier in those with inadequate health infrastructures, less capacity to cope with epidemics, and limited healthcare budgets.

Q2. Does the ranking of the countries based on the new SI differ from the individual ICRI and FII rankings?

By applying the multi-criteria decision-making technique TOPSIS, we develop an SI that can be used to establish a ranking covering all the dimensions of the ICRI and the FII. The 112 countries analysed are ranked from highest to lowest joint risk of food insecurity and structural risk factors for COVID-19 impacts, thereby providing an answer to Q2 (Table 5 of the appendix). Focusing the analysis on the extreme values, we carry out a detailed study of the first and last 20 positions of the ranking in an effort to identify similarities between the proposed SI and the indices used to construct it, ICRI and FII (Table 3 shows countries with the highest risk and Table 4 those with the lowest risk).

**Table 3:** Comparison of the top 20 positions of the SI compared to the FII and ICRI

COUNTRIES	Ranking IS	Ranking FII	Ranking ICRI
Haiti	1	9	3
Zambia	2	3	11
Burundi	3	7	4
Madagascar	4	8	8
Chad	5	11	1
Sierra Leone	6	6	7
Malawi	7	4	18
Yemen	8	1	5
Togo	9	21	16
Congo	10	16	2
Mozambique	11	15	9
Ethiopia	12	5	13
Guinea	13	12	14
Nigeria	14	14	15
Sudan	15	2	17
Burkina Faso	16	26	6
Benin	17	22	19
Uganda	18	19	12
Angola	19	17	22
Laos	20	23	38

**Table 4:** Comparison of the bottom 20 positions of the SI compared to the FII and ICRI

COUNTRIES	Ranking IS	Ranking FII	Ranking ICRI
Malaysia	93	71	82
France	94	96	90
Russia	95	89	84
Oman	96	80	88
Germany	97	99	104
Denmark	98	97	107
Singapore	99	94	100
UAE	100	72	99
Ireland	101	111	92
Netherlands	102	110	102
United Kingdom	103	107	98
Australia	104	83	106
Sweden	105	106	105
United States	106	102	101
Finland	107	112	109
Israel	108	105	97
Norway	109	95	112
Canada	110	101	110
New Zealand	111	100	108
Switzerland	112	103	111

As can be seen in Table 3, most of the countries that occupy the top positions in the SI are in Africa with the exception of two Asian countries (Yemen and Laos) and the top country in the ranking, Haiti, which is Caribbean. These are countries that are afflicted by extreme poverty, which influences both the quality of their food and their capacity to respond to the pandemic. In this vein, Éliás and Jámbor (2021) conclude that low income is the factor that has the strongest negative effect on food security during the COVID-19 pandemic. Likewise, Erokhin and Gao (2020) demonstrate that in these countries the level of food security is mainly related to economic access to an adequate supply of food, while in higher income countries it is linked to the availability of food.

Haiti has been ranked first in terms of the combined risk. According to Louis-Jean et al. (2020), it is a country with limited medical supplies, infrastructure and health professionals, and as such is extremely vulnerable to coronavirus. Haitians are aware of the risks and preventive measures needed to combat the virus, yet the majority of the population still does not have the essential tools and equipment to deal with it. In Haiti, 70% of households live in chronic poverty, only 36% of the population has access to electricity, around 56% to safe drinking water, and 28% to basic sanitation. The situation in the country is very difficult, although the ranking points to similar circumstances in low-income countries, such as Zambia and Burundi, among others.

Chad, which holds the top spot in the ICRI and fifth in the SI, is a country where 33.7% of the population lives in extreme poverty. Its government has imposed measures that limit mobility and social and religious gatherings, while creating a fund called "FS Covid-19" aimed at reinforcing medical infrastructure and its functionalities, enabling a rapid response to the needs of health centres (Tchana et al., 2020). Given the lack of resources, if no action is taken, hospitals that treat infected people could quickly become hotbeds of infection (Cénat, 2020).

Yemen ranks first in the FII ranking and eighth in the SI. This country is rated as one of the poorest in the world, with structural vulnerabilities that have developed over a prolonged period of conflict and poor governance, and where more than 50% of the population is facing famine (Tandon & Vishwanath, 2020). As pointed out by Hashim et al. (2021), Yemen is facing a triple emergency with the decline in humanitarian aid, the ongoing war and the pandemic. The authors conclude that the action most urgently needed is to request that local authorities join forces in tackling the COVID-19 health situation by limiting their interference and delivering essential humanitarian aid.

From a global perspective of the top 20 positions, the results of the SI ranking reveal the relationship between the structural risk factors for COVID-19 impacts and food insecurity, with the top 20 countries according to the SI ranking also holding the top 20 positions in the individual rankings, thus providing a negative answer to Q2. However, at the individual level, Yemen and Sudan, which occupied the top positions for food insecurity, drop to 8th and 15th positions, respectively, in the SI. Countries such as Haiti and Zambia are in a more worrying situation in this regard (1st and 2nd place in the SI, respectively).

The analysis of the countries at lower risk is carried out below, focusing on the bottom 20 of the 112 countries in the sample (Table 4).

The group of 20 countries with the lowest overall risk of food insecurity and lack of resources to tackle COVID-19 is more geographically dispersed, being composed of 11 nations in Europe, 5 in Asia, 2 in Oceania and 2 in North America. They are developed economies with sufficient resources to alleviate a critical situation of food insecurity and a pandemic. Switzerland is the country with the lowest overall risk, holding the last position in the SI, which is better than its position in the two individual indices (103rd in the FII ranking and 111th in the ICRI).

In Europe, the pandemic has given rise to anomalous situations: images of empty supermarket shelves, shortages of farm workers, an increase in the use of food banks, as well as greater concern for the health of people at the lower end of the socio-economic scale (Ranta & Mulrooney, 2021), where food insecurity has had a more intense impact. However, Matthews (2020) concluded that EU farmers, processors and retailers have maintained food supplies to EU consumers and adjusted to the shift in demand caused by the lockdowns in the food service sector. In this context, Capodistrias et al (2022) examines the impact of the COVID-19 crisis on the functioning of European food banks, and their results indicated that food banks were able to redistribute a significantly higher amount of food despite numerous social restrictions and other challenges associated with the pandemic.

In short, the group of 20 countries with the lowest combined risk (food insecurity and COVID-19 impacts), are also ranked between 96 and 112th for the FII (except Malaysia, Oman, UAE and Norway) and the ICRI (except Malaysia, France, Russia, Oman and Ireland). Therefore, the answer to Q2 is again negative for this set of countries, although it is important to note that Switzerland occupies the position of lowest risk with the new SI.

## Conclusions

This paper analyses two major problems that are widespread in the world's economies: the structural risk factors for adverse impacts of COVID-19 and food insecurity. The aim of the research is to identify whether the two problems are related, with the study covering a wide range of developed and developing countries in 2020. The overall scores for the FII and the ICRI demonstrate that countries with greater food insecurity are more at risk of lacking resources to cope with COVID-19, with emerging economies at the upper end of the ranking (such as Haiti, Chad or Yemen) and developed economies at the lower end (e.g. Finland, Switzerland or Sweden).

Pairwise comparison of the FII and ICRI reveals a significant degree of association (according to the *χ*^2^ test of the contingency tables), and in most cases a positive relationship. We thus find further evidence that the two problems are connected. This indicates to international organizations that countries with worse food insecurity will suffer more serious consequences due to extreme situations such as the one experienced during the pandemic, and thus have a greater need for funding.

Lastly, the development of an SI with seven indicators sourced from the pillars of the FII and the ICRI confirms that the countries with the worst food insecurity are those facing the greatest structural risk of adverse impacts of COVID-19. However, the ranking directs international organizations' attention to countries such as Haiti, Zambia and Burundi and their greater need for an injection of financial aid than other emerging countries. Conversely, Switzerland is the country with the lowest combined risk. So, the health crisis has exacerbated the vulnerability of its low-income population to a lesser degree than other countries.

The indexes used in the research have a number of limitations. FII does describe the food security conditions. However, it does not measure the outcomes of food security, namely food consumption or malnutrition figures. Also, the index namely focuses on the GDP as well as poverty and on the agricultural production side. The FII extends to governance and policy areas that are usually not directly included in food security indicators. It is thus complementary to other food security measures, but it is not a substitute. The ICRI is focused on structural factors. It does not contain rapidly changing information, for example on cases, government restrictions, and changing health system capacity in response to the pandemic. There is a temporal imbalance between the long-standing food insecurity in certain areas and structural risk factors in the face of a pandemic, as the latter is in theory a more short-lived problem. Thus, the research is limited to the analysis of one year, 2020, due to the lack of information for more periods, and it uses statistical techniques appropriate for the existing data. In the longer term, it will be possible to identify how the most at-risk countries have been able to solve the problems concerning the food system and the struggle to overcome a pandemic situation.

## Appendix

See Appendix Tables

**Table 5:** Contingency tables for pairs of pillars

Hazard and exposure	Affordability					Vulnerability	Affordability				
	Very good	Good	Moderate	Weak	Total		Very good	Good	Moderate	Weak	Total
Very high	0	13	6	0	20	Very high	20	0	0	2	22
High	3	1	9	1	14	High	9	0	0	8	17
Medium	6	0	8	19	33	Medium	8	3	10	19	40
Low	30	2	1	12	45	Low	2	13	14	3	33
Total	39	16	24	32	112	Total	39	16	24	32	112

Lack of coping capacity	Affordability					Hazard and exposure	Availability				
	Very good	Good	Moderate	Weak	Total		Very good	Good	Moderate	Weak	Total
Very high	0	4	7	2	13	Very high	0	6	14	0	20
High	2	5	13	7	28	High	0	1	11	2	14
Medium	2	7	3	11	23	Medium	0	0	16	17	33
Low	12	0	1	9	22	Low	1	1	11	32	45
Very low	23	0	0	3	26	Very low	0	0	0	0	0
Total	39	16	24	32	112	Total	1	8	52	51	112

Vulnerability	Availability					Lack of coping capacity	Availability				
	Very good	Good	Moderate	Weak	Total		Very good	Good	Moderate	Weak	Total
Very high	1	0	4	17	22	Very high	0	4	8	1	13
High	0	0	4	13	17	High	0	3	22	3	28
Medium	0	2	19	19	40	Medium	0	1	14	8	23
Low	0	6	25	2	33	Low	0	0	4	18	22
Very low	0	0	0	0	0	Very low	1	0	4	21	26
Total	1	8	52	51	112	Total	1	8	52	51	112

Vulnerability	Quality and safety					Lack of coping capacity	Quality and safety				
	Very good	Good	Moderate	Weak	Total		Very good	Good	Moderate	Weak	Total
Very high	19	0	0	3	22	Very high	0	3	7	3	13
High	9	0	2	6	17	High	1	3	18	6	28
Medium	9	1	13	17	40	Medium	4	2	9	8	23
Low	2	7	20	4	33	Low	11	0	1	10	22
Very low	0	0	0	0	0	Very low	23	0	0	3	26
Total	39	8	35	30	112	Total	39	8	35	30	112

Hazard and exposure	Quality and safety					Hazard and exposure	Natural resources and resilience			
	Very good	Good	Moderate	Weak	Total		Good	Moderate	Weak	Total
Very high	0	7	13	0	20	Very high	3	17	0	20
High	2	1	9	2	14	High	3	11	0	14
Medium	6	0	10	17	33	Medium	6	25	2	33
Low	31	0	3	11	45	Low	1	34	10	45
Total	39	8	35	30	112	Total	13	87	12	112

Vulnerability	Natural resources and resilience				Lack of coping capacity	Natural resources and resilience			
	Good	Moderate	Weak	Total		Good	Moderate	Weak	Total
Very high	0	17	5	22	Very high	2	11	0	13
High	0	13	4	17	High	2	26	0	28
Medium	7	30	3	40	Medium	5	18	0	23
Low	6	27	0	33	Low	3	13	6	22
Very low	0	0	0	0	Very low	1	19	6	26
Total	13	87	12	112	Total	13	87	12	112

^5^ and

**Table 6:** Ranking of all the countries analysed

COUNTRIES	Ranking IS	Ranking FII	Ranking ICRI
Haiti	1	9	3
Zambia	2	3	11
Burundi	3	7	4
Madagascar	4	8	8
Chad	5	11	1
Sierra Leone	6	6	7
Malawi	7	4	18
Yemen	8	1	5
Togo	9	21	16
Congo	10	16	2
Mozambique	11	15	9
Ethiopia	12	5	13
Guinea	13	12	14
Nigeria	14	14	15
Sudan	15	2	17
Burkina Faso	16	26	6
Benin	17	22	19
Uganda	18	19	12
Angola	19	17	22
Laos	20	23	38
Niger	21	27	10
Cameroon	22	20	21
Rwanda	23	10	30
Tanzania	24	25	23
Bangladesh	25	30	28
Côte d'Ivoire	26	32	25
Nepal	27	37	29
Senegal	28	24	27
Pakistan	29	34	26
Syria	30	13	37
Kenya	31	28	24
Venezuela	32	18	45
Cambodia	33	33	31
Uzbekistan	34	31	54
Tajikistan	35	29	46
Botswana	36	40	39
Mali	37	35	20
Honduras	38	47	32
Sri Lanka	39	39	47
Ghana	40	36	34
Guatemala	41	42	35
Nicaragua	42	38	43
Philippines	43	41	40
Bolivia	44	50	33
India	45	43	42
Myanmar	46	44	36
Indonesia	47	49	44
South Africa	48	45	41
El Salvador	49	48	52
Ukraine	50	60	56
Jordan	51	52	48
Paraguay	52	53	69
Dominican Rep	53	66	50
Ecuador	54	46	57
Tunisia	55	55	62
Vietnam	56	51	61
Morocco	57	57	53
Azerbaijan	58	58	55
Peru	59	68	51
Serbia	60	62	71
Colombia	61	61	49
Egypt	62	54	70
Thailand	63	63	63
Bulgaria	64	70	68
Argentina	65	59	74
Algeria	66	56	58
Bahrain	67	65	59
Belarus	68	90	66
Hungary	69	78	76
Panama	70	73	64
Slovakia	71	74	79
Turkey	72	67	60
Kuwait	73	81	65
Qatar	74	77	80
Greece	75	86	81
Romania	76	91	72
Poland	77	88	87
Czech Rep	78	108	78
Mexico	79	69	67
Uruguay	80	84	83
Kazakhstan	81	82	89
Brazil	82	64	73
Portugal	83	93	96
Italy	84	98	85
China	85	75	75
Saudi Arabia	86	76	93
Austria	87	109	91
Spain	88	87	94
Japan	89	104	103
Chile	90	79	86
Belgium	91	92	95
Costa Rica	92	85	77
Malaysia	93	71	82
France	94	96	90
Russia	95	89	84
Oman	96	80	88
Germany	97	99	104
Denmark	98	97	107
Singapore	99	94	100
U. Arab Emirates	100	72	99
Ireland	101	111	92
Netherlands	102	110	102
United Kingdom	103	107	98
Australia	104	83	106
Sweden	105	106	105
United States	106	102	101
Finland	107	112	109
Israel	108	105	97
Norway	109	95	112
Canada	110	101	110
New Zealand	111	100	108
Switzerland	112	103	111

^6^.

## Footnotes

1 Table 1A in the appendix contains all the contingency tables with the absolute frequency of the countries.
